# The Engineering Side of Hospital Work

**Published:** 1905-12-30

**Authors:** 


### THE ENGINEERING SIDE OF HOSPITAL WORK.

### XVI.—APPARATUS FOR GENERATING ELECTRICITY.

(*Continued from page* 172.)

#### The Difference between Continuous Current and Alternate Current Generators.

The whole difference lies in the method of collecting the currents that are generated, and in supplying the currents for the field magnets coils. In all dynamo machines the currents are alternate in direction, when they are generated, but in the continuous current apparatus they are collected before they change their direction, and in the alternate current generators they are collected just as they are created. Alternate currents also cannot be used for the coils of the field magnets, since the magnetism they create is constantly changing, while the current generated by continuous currents is used for the field magnets of the dynamo which generates them. For the collection of the currents in continuous current machines, what is termed a commutator is added to the armature, consisting of a cylinder of copper built up of sectors divided from each other by plates of mica, the whole being held together mechanically. The conductors on the armature are wound in coils corresponding in number to the sections of the commutator, and the two adjacent ends of each two adjacent coils are connected to one of the commutator segments. The commutator is carried on the same shaft as the armature, but is carefully insulated, so that the only possible connection, so long as all is in order, between the commutator and any part of the armature, is by way of the coil ends. Brushes, as they are called from the fact that the early forms were brushes of copper wire, but now more usually slabs of carbon, bear on the commutator at certain points, there being as many brushes, or groups of brushes, as there are magnet poles. The brushes are carried by a brass or iron ring, which is usually fixed on the bearing of the armature axle, and is capable of being turned round on it, and the position of the brushes is that where the direction of the current generated is on the point of reversal. The electric circuit is actually broken and re-made at each brush as each coil passes under it, and there is a spark passing between the brush and the commutator, which gradually wears away both. In large machines there are groups of brushes at each collecting point, each brush being held in a shoe or other device, with a spring pressing it on the commutator surface, the shoes at each collecting point being carried on a brass spindle attached to the main carrier. The spindles carrying the brush shoes are insulated from the main carrier, and those of the same name, those generating currents in the same direction, are connected together by copper rings or other convenient arrangement, so that all the currents in the positive direction are collected from one terminal, and all the negative from another. With alternate current machines, where the armature revolves, two, three, or four rings are fixed on the axle of the armature, insulated from it, and connected to the free ends of the armature coils. The whole of the armature coils, in single-phase alternate current machines, are in one length, with two free ends, and the two sets of coils of the two-phase machines have two free ends to each set, and these are connected to two sets of collecting rings. With three-phase machines, with revolving armatures, there are two arrangements. In one there are three free ends, which are connected to the three collector rings on the shaft, the other three ends of the three sets of coils being connected together, and sometimes to a fourth collector ring. In another form of three-phase and two-phase machine, where the armature revolves, the armature coils are wound as in a continuous current machine, in slots on its surface, but there is no commutator, and there are no breaks in the coils. For three-phase working three points in the coils 180 degrees apart in the periphery of the armature are tapped, the tappings being connected to the three collector rings. For two-phase, four points are tapped, 90 degrees apart, the tappings being connected to the four rings. The current in the coils of the field magnets is provided by a separate machine, sometimes driven by ropes from a pulley on one side of the generator, and sometimes the little machine is mounted on the frame of the larger one, the axles of the two being connected together, and revolving together, the machine supplying its own field magnet current. Where there are a number of alternators together, one machine may supply current for the whole. This plan is also adopted with continuous current machines where there are a number together. Where the armature is stationary, the coils being on the inner side of the outer cylinder, their ends are merely brought out to terminals on the frame, the field magnets being supplied either by a little dynamo carried on the frame of the large machine, as explained, or by current from a separate machine. There is one other point in connection with alternate current machines—the number of alternations, and how they are produced. The number of alternations is produced by the number of fields through which the armature conductors pass, multiplied by the number of times the conductors pass through them—that is, by the number of revolutions per second or per minute. With machines furnishing alternate currents having 50 periods, or alternations, per second or 3,000 per minute, and running, say, at 600 revolutions per minute, only five complete magnetic fields are necessary, or if the machine runs at 500 revolutions, six magnetic fields are necessary. Many alternate current machines are constructed to furnish currents at 50 and 60 periods, the higher number being obtained by driving the machine faster. Another point had also better be explained, why a certain number of alternations are necessary. This is because the current is used for lighting, and when that is so the number of alternations must be sufficient to mask the inequalities that would be otherwise visible in the light given. As already explained, the alternate current is continually rising and falling and reversing, and the heat generated by the current in the filament of the incandescent lamp, unless the latter be of a large section, or the alternation very rapid, as in the case of high candle power lamps, varies with the variations in the current, the light being good when the current is at its maximum, and lessening as the current weakens. The strongest illustration of this is where a dynamo is run with one of the gas engines of the older pattern, by a single cylinder steam engine with a very heavy crank, or by a belt with a badly made splice. With the gas engine, at the explosion, the pressure generated by the dynamo is suddenly increased, and then gradually lowers, to be increased at the next explosion, and so on. With single-cylinder engines with heavy cranks, and with belt-driven dynamos, with badly made splices, the same thing happens, the pressure is increased at a certain moment, and then decreases. The result is a painful wink in the lights. At one of the exhibitions in the early days of electric lighting it was possible to count the strokes of the engine driving a particular dynamo, in the lamps the dynamo was supplying in the grounds a long way off. If either the filament is increased in section, or the currents are made to follow each other very rapidly, the wink disappears, the heating effect being reduced to an average when once the filament is heated up. You may take almost what liberties you please with a 300 candle power incandescent lamp in the matter of variation of pressure, and the same applies to rapid alternations. The minimum number that is not visible is 25 per second, but even these may be with very thin filaments, and hence at the present time practice has settled to 50 periods for town supply and similar work. The smaller the number of alternations the better for efficiency, as the constant changes and reversals lead to inductive actions, which waste energy approximately in proportion to the number of alternations.

### Currents Supplied by Town Generating Stations.

The currents supplied by electricity supply stations in towns are generated in the manner described above, and are conveyed to consumers by cables under the streets usually. They have all the characteristics that have been mentioned, and, in addition, they have one characteristic that is absent where the current is generated in the hospital itself—the pressure at which it is delivered is beyond the control of the hospital engineers. In the first place, the service pressure is the only one available without special arrangement. That is to say, the pressures at which town generating stations are now supplying varies from 200 volts to 260 volts for incandescent lamps, with double those figures for motors, and whatever the figure is, the hospital that requires current must take it at that figure, unless special arrangements for transformation are made. Occasionally, where the consumption is large, the current is brought to the hospital at a much higher pressure, and is transformed down there, but the same thing holds—the final pressure must be that of the station. But this is the nominal pressure. In practice it may be above or below this, according to the exigencies of the generating station. If there has been a heavy increase in the demand in the neighbourhood of the hospital, the pressure may go down very considerably, and *vice versâ*. It is as well for members of the medical profession to remember this, as the efficient working of some of their apparatus may be seriously affected by variations of pressure, and the matter is absolutely beyond their control. On the other hand, provided that proper apparatus is available, any current, at any pressure, supplied by the town generating stations can be converted to any other current, and to any form of electricity, no matter what the variations are, and kept under control, provided the engineer is given the apparatus he requires and is allowed to use them.

